# Association between divorce and access to healthcare services among married immigrants: propensity score approaches

**DOI:** 10.1186/s13690-022-00840-3

**Published:** 2022-03-14

**Authors:** Suyeong Bae, James E. Graham, Sanghun Nam, Ickpyo Hong

**Affiliations:** 1 Department of Occupational Therapy, Graduate School, Yonsei University, Wonju-si, Gangwon-do Republic of Korea; 2 Department of Occupational Therapy, Colorado State University, Fort Collins, CO USA; 3 Department of Occupational Therapy, College of Software and Digital Healthcare Convergence, Yonsei University, Wonju-si, Gangwon-do Republic of Korea

**Keywords:** Immigrants, Divorce, Health services accessibility, Health services research

## Abstract

**Background:**

While divorce is a social determinant of health among married immigrants in Korea, its association with access to healthcare services is unclear. Given the rapid increase in the number of married immigrants in Korea, research is needed to improve minority groups’ access to healthcare services. Here, we examined healthcare service utilization among married immigrants.

**Methods:**

We retrieved 11,778 adults from the 2018 Korea National Multicultural Family Survey. We analyzed whether the sex of divorced immigrants is associated with healthcare access using multivariable logistic regression analysis. Further, we analyzed the association between divorce and access to healthcare services among married immigrants using propensity score matching methods.

**Results:**

There were 691 (5.8%) divorced immigrants in the data set. The married male immigrants had no association between divorce status and healthcare access (adjusted odds ratio [OR] = 1.05, 95% confidence interval [CI] = 0.55–2.03, *p* = 0.8620). Divorced immigrants were less likely to receive healthcare services than married immigrants (adjusted OR = 1.42, 95% CI = 1.07–1.88).

**Conclusion:**

Our findings revealed that divorce increases the risk of limited access to healthcare services among married immigrants. Policymakers and healthcare providers should be aware of these potential disparities in this vulnerable minority population.

## Background

The persistent demand for labor and capital has greatly increased the national mobility of the population. The influx of foreigners into Korea began increasing in the 1990s and continues up to this day [1]. There are several types of immigrants, including those who are coming for international studies, employment, and marriage. Each type of immigrant has distinct characteristics. Notably, unlike those studying abroad or engaged in international employment, those coming for marriage (or “married immigrants”) typically want to become permanent residents [2]. These characteristics have increased the need for specific immigration policies and the welfare of married immigrants.

The health of married immigrants worldwide has become a distinct field of study [3]. Immigrants must live a new life by adapting to a socio-cultural environment that differs from their home country. They experience various challenges such as language problems, economic difficulties, and cultural conflicts [4]. In addition, their health may deteriorate because of difficulties in understanding health insurance and the healthcare system [5]. This can reduce the health of married immigrants and lead to poor health literacy.

Health literacy guides individuals to understand and use information to promote and maintain health. A decline in health literacy is associated with poor disease management. This results in lower health levels and higher healthcare expenditures, as health literacy of married immigrants is a major factor that influences access to health-related services [6].

The number of married immigrants entering Korea is increasing, and the country is rapidly becoming a multicultural society. According to the 2019 immigration statistics released by the Korea Immigration Service, the number of married immigrants in 2019 was 166,025, an increase of 14,417 from 2015, and an average increase of 3.3% in the last 3 years [7]. To prepare for a multicultural society, Korea has increased the number of welfare policies and educational programs for married immigrants [8]. For instance, depression has increased, and also life satisfaction among married female immigrants has decreased [9, 10]. Married female immigrants living in Korea are also more likely to experience health inequality [11]. In addition, studies on the health-related quality of life and use of healthcare services of married immigrants only target married female immigrants; to the best of our knowledge, there is limited knowledge on the current status of the health of married male immigrants.

As the number of married immigrants is increasing, divorce is becoming a common phenomenon. According to the 2019 Marriage and Divorce Statistics Report, the divorces of married immigrants accounted for 6.2% of all divorces [7]. Divorce is a social determinant of one’s health. Indeed, research on the effect of changing marital status on concomitant changes in health behavior reveals that health disabilities and changes in health promotion were evident in women who experienced divorce or death of a spouse [12]. For married immigrants, divorce leads to social isolation and has a profound impact on their lives [13]. As changes in life may worsen health indicators, we need to understand how divorce affects an individual’s health. However, the link between changes in marital status and changes in health behaviors among married immigrants is unclear.

Our study addresses the limitations of previous studies. First, most studies on married immigrants have been limited to women. According to Statistics Korea, the 2019 population of married male immigrants was 28,931; however, studies including married male immigrants in the literature review were insufficient [7]. Second, divorce is directly related to economic problems, but its relationship with access to healthcare services is unclear. Therefore, this study aims to examine the association between divorce and access to healthcare services among married male and female immigrants while accounting for various socioeconomic factors.

## Methods

### Study population

We retrieved the initial sample of 17,073 adults from the 2018 Korea National Multicultural Family Survey. Our final sample included 11,778 ever married immigrants after excluding 5295 cases with missing values on the study variables. The Korea National Multicultural Family Survey is conducted by the Korea Ministry of Gender Equality and Family and targets every married immigrant, their spouses and children. It is implemented to understand the current status of family relations, lifestyles, family problems, economic conditions, and educational support for multicultural families. In this study, the de-identified data were retrieved, and the study has been reviewed and approved by the Yonsei University Institutional Review Board.

### Study variables

#### Independent and dependent variables

The independent variable was divorce status (yes or no) among married immigrants. The dependent variable was difficulty in accessing healthcare services defined by the question, “Did you have any experiences in the past year where you could not go to the hospital (e.g., inpatient facilities or ambulatory care) when you were sick?” [yes, no].

#### Confounders

The confounders were demographics, self-reported social behavior, and economic status characteristics, including experiences participating in meetings, frequency of social services experience, social discrimination, socioeconomic status, sadness or despair, life satisfaction, and self-rated health status (Table 1). The experiences of participating in meetings were divided into various sub-themes such as parents, friends, community society, religion, and civic group meetings. The frequency of social service experience was the number of times an individual received support on economic and psychological consultation from the government agencies. Social discrimination was assessed based on whether the survey participants experienced any type of discrimination in the local community (yes or no). Life satisfaction and self-rated health status were measured using a 5-point Likert scale (1 = very good, 2 = good, 3 = neutral, 4 = poor, and 5 = very poor). Socioeconomic status was measured using a 3-point Likert scale (1 = high, 2 = middle, and 3 = low). Sadness or despair was used to assess the emotional status as a dichotomous category (yes, no).

**Table 1:** Demographic information of participants before and after 1:1 greedy propensity score matching

Variables	Before matching (*N* = 11,778)			After matching (*N* = 1364)		
	No divorce (*n* = 11,087)	Yes divorce (*n* = 691)	*p*	No divorce (*n* = 682)	Yes divorce (*n* = 682)	*p*
Age ^a^ (years)	39.12 (10.50)	45.14 (10.93)	0.1367	45.39 (12.77)	45.09 (10.89)	0.6369
Sex			0.0981			0.4649
Male	1992 (17.97)	107 (15.48)		117(17.16)	107 (15.69)	
Female	9095 (82.03)	584 (84.52)		564 (82.84)	575 (84.31)	
Currently employed (Yes)	6733 (60.73)	535 (77.42)	<.0001^*^	525 (76.98)	526 (77.13)	0.9487
Educational attainment			0.0346^*^			0.8556
Less than Elementary school	9693 (87.43)	623 (90.16)		616 (90.32)	614 (90.03)	
Above Middle school	1394 (12.57)	68 (9.84)		66 (9.68)	68 (9.97)	
Residential area			<.0001^*^			0.8497
Urban	7078 (63.84)	527 (76.27)		515 (75.51)	518 (75.95)	
Suburban	4009 (36.16)	164 (23.73)		167 (24.49)	164 (24.05)	
Household income (₩)			<.0001^*^			0.7500
< 1,000,000	347 (3.13)	164 (23.73)		152 (22.29)	155 (22.73)	
1,000,000–3,000,000	4292 (38.71)	455 (65.85)		465 (68.18)	455 (66.72)	
3,000,000–5,000,000	4647 (41.91)	62 (8.97)		59 (8.65)	62 (9.09)	
> 5,000,000	1801 (16.24)	10 (1.45)		6 (0.88)	10 (1.47)	
Difficulties in living in Korea (Count)			0.0017^*^			0.6510
1	5471 (49.35)	305 (44.14)		319 (46.77)	302 (44.28)	
2	2922 (26.36)	224 (32.42)		211 (30.94)	220 (32.26)	
3	2694 (24.30)	162 (23.44)		152 (22.29)	160 (23.46)	
Participation activities (Yes)						
Parents	1645 (14.84)	53 (7.67)	<.0001^*^	61 (8.94)	53 (7.77)	0.7277
Friends	6621 (59.72)	336 (48.63)	<.0001^*^	339 (49.71)	335 (49.12)	0.8285
Community	1408 (12.70)	37 (5.35)	<.0001^*^	37 (5.43)	37 (5.43)	1.0000
Religion	2698 (24.33)	127 (18.38)	0.0004^*^	146 (21.41)	125 (18.33)	0.1541
Civic groups	701 (6.32)	21 (3.04)	0.0005^*^	17 (2.49)	20 (2.93)	0.6171
Experiences of social discrimination (Yes)	3513 (31.69)	208 (30.10)	0.3847	200 (29.33)	208 (30.50)	0.6362
Self-rated health			<.0001^*^			0.5191
Very Good	3139 (28.31)	101 (14.62)		103 (15.10)	101 (14.81)	
Good	5206 (46.96)	246 (35.60)		262 (38.42)	245 (35.92)	
Neutral	2121 (19.13)	174 (25.18)		182 (26.69)	174 (25.51)	
Poor	555 (5.01)	142 (20.55)		111 (3.52)	134 (19.65)	
Very Poor	66 (0.60)	28 (4.05)		24 (3.52)	28 (4.11)	
Sadness or despair (Yes)	2867 (25.86)	331 (47.90)	<.0001^*^	316 (46.33)	324 (47.51)	0.6643
Socioeconomic status			<.0001^*^			0.8288
High	706 (6.37)	13 (1.88)		14 (2.05)	13 (1.91)	
Middle	7594 (68.49)	204 (29.52)		193 (28.30)	203 (29.77)	
Low	2787 (25.14)	474 (68.60)		475 (69.65)	466 (68.33)	
Life satisfaction			<.0001^*^			0.7548
Very Good	3248 (29.30)	50 (7.24)		49 (7.18)	50 (7.33)	
Good	4065 (36.66)	165 (23.88)		165 (24.19)	164 (24.05)	
Neutral	3092 (27.89)	294 (42.55)		307 (45.01)	290 (42.52)	
Poor	615 (5.55)	160 (23.15)		146 (21.41)	157 (23.02)	
Very Poor	67 (0.60)	22 (3.18)		15 (2.20)	21 (3.08)	
Government support (Yes)	319 (2.88)	87 (12.59)	<.0001^*^	93 (13.64)	85 (12.46)	0.5202
Frequency of social services experience ^a^	1.86 (2.04)	1.27 (1.72)	<.0001^*^	1.24 (1.70)	1.28 (1.72)	0.8118

Values are presented as mean (standard deviation) or numbers (%)

^a^ Mean (standard deviation)

^*^p < 0.05

#### Data analysis

The study used a retrospective cross-sectional design based on a nationally representative survey database. Descriptive statistics (Chi-squared test and Wilcoxon rank-sum nonparametric test) were used to compare the characteristics of the two comparison groups (divorce vs. non-divorce). Categorical variables were presented as frequency and percentage, whereas numeric variables were presented as mean and standard deviation. We first analyzed whether the sex of divorced immigrants was associated with healthcare access using multivariable logistic regression analysis (divorced male vs. divorced female immigrants). Then, we analyzed whether the divorced status of married immigrants is associated with healthcare access. To assess the robustness of the point estimates, we used three confounders adjustment methods: one based on multivariate logistic regression, and two based on propensity score matching analyses. Regarding the matching methods, we specifically used inverse probability of treatment weighting (IPTW) with average treatment effect (ATE) weight and 1:1 greedy propensity score matching [14].

The propensity score was calculated using logistic regression analysis that accounted for the study confounders. The most common propensity score matching approaches are 1:1 greedy propensity score matching and IPTW [14]. Divorced and non-divorced immigrant groups were matched based on a propensity score that reflected the distribution of the study confounders. IPTW with ATE was weighted to calculate a generalized estimate of the entire population from which the observed samples were drawn [15]. For the 1:1 greedy propensity score matching approach, we performed diagnostics and checked if the study confounders were balanced across the two comparison groups using the logit propensity score (LPS). LPS represents how well the distribution of variables is balanced [16]. We considered a value between − 0.1 and 0.1 standardized mean difference of all confounders, and LPS (*p* > 0.05) as a good balance by the 1:1 greedy propensity score matching [17]. The statistical significance level was set at *p* < 0.05. We calculated the adjusted odds ratio (AOR) as a point estimation with 95% confidence interval (CI). All analyses were performed using the SAS version 9.4 software.

## Results

The final sample included 691 (5.9%) divorced and 11,087 (94.1%) non-divorced immigrants. The average age of divorced immigrants was 45.1 years (standard deviation [SD] = 10.9) and that of non-divorced immigrants was 39.1 years (SD = 10.5). Table 1 summarizes the group differences before and after 1:1 greedy propensity score matching. Before matching, 16 of the 19 variables were statistically different between the two groups (all *p* < 0.05). After matching, all variables and LPS were balanced between the two comparison groups (all *p* > 0.05); further, the absolute values of the standardized mean differences between divorced and non-divorced immigrants were less than 0.10 [17].

Table 2 shows the results of the association between divorce status and access to healthcare according to the sex of married immigrants. For married female immigrants, divorce status and healthcare access were associated with access to healthcare (AOR = 1.414, 95% CI = 1.089–1.836, *p* = 0.0094), but not for married male immigrants (AOR = 1.059, 95% CI = 0.552–2.033, *p* = 0.8620). Thus, the association between divorce status and access to healthcare differed by sex.

**Table 2:** Association between sex and difficulties in healthcare services access for divorced immigrants

Estimated method	Limited healthcare services access					
	Male			Female		
	AOR^†^	95% CI	*p*	AOR^†^	95% CI	*p*
Adjusted logistic regression	1.059	0.552–2.033	0.8620	1.414	1.089–1.836	0.0094^*^

*AOR* adjusted odds ratio, *CI* confidence interval

^†^ *p*-values for estimated odds ratios were calculated using multivariable logistic regression

^*^p < 0.01

Table 3 presents the association between divorce and difficulties in using healthcare services. In the unadjusted model, divorced immigrants exhibited higher risk of experiencing difficulty in accessing healthcare services than non-divorced immigrants (odds ratio [OR] = 2.323, 95% CI = 1.910–2.826). While the magnitude of risk decreased in the adjusted model (AOR = 1.367, 95% CI = 1.076–1.736) and the two propensity score matching models (IPTW: AOR = 1.147, 95% CI = 1.057–1.243; 1:1 greedy matching: AOR = 1.423, 95% CI = 1.075–1.882; Table 3), the point estimations remained statistically significant (all *p* < 0.05).

**Table 3:** Association between divorce and difficulties in healthcare services access

Estimated method	Limited healthcare services access		
	AOR^†^	95% CI	*p*
Unadjusted logistic regression	2.323	1.910–2.826	<.0001^**^
Adjusted logistic regression	1.367	1.076–1.736	0.0103^*^
Inverse probability of treatment weighting and ATE weight with logistic regression	1.147	1.057–1.243	0.0009^**^
1:1 Greedy propensity score matching with logistic regression	1.423	1.075–1.882	0.0135^*^

*AOR* adjusted odds ratio, *CI* confidence interval, *ATE* average treatment effect

^†^ *p*-values for estimated odds ratios were calculated using multivariable logistic regression

^*^p < 0.05, ^**^ *p* < 0.01

## Discussion

We investigated the association of divorce status with married immigrants’ access to healthcare services by sex. Further, we compared the use of healthcare services by married immigrants according to divorce status. Healthcare service usage differed for married female immigrants according to their divorce status, but not for married male immigrants. Divorced immigrants were more likely to not use healthcare services than non-divorced immigrants. Importantly, our findings could inform clinicians and healthcare policymakers about critical implications for divorced versus non-divorced immigrants’ use of healthcare services.

The number of married immigrants residing in Korea is increasing, along with the divorce rate among them [7]. Divorce is a social determinant of health and has a profound impact on the lives of married immigrants [13]. Research shows that divorce affects the lives of married female immigrants. Notably, their life satisfaction was low; moreover, because of their low income, they experienced difficulty in social adaptation [18]. However, to the best of our knowledge, there is limited research on the association between divorce and healthcare service usage.

Our study attempted to fill this research gap. We find an association between the divorce of married immigrants and their usage of healthcare services. Social determinants of health are important factors for improving the quality of public health [19]. Public health should prevent diseases and promote health; to this end, we need to consider the background and environment of individuals [20]. However, our results suggest an insufficiency of public health policies for married immigrants. Further, our results point to the need for an in-depth study on the effects of the social determinants of married immigrants’ health on public health, and the necessity of establishing a holistic and comprehensive health policy.

Social determinants of health emphasize the importance of the environment in public health [21]. Health-related behavior is influenced by the environment; therefore, an individual’s health may vary depending on the environment [22]. Therefore, it is important to determine how the environment can be modified. For example, the quantity and quality of various social relationships, as well as employment play important roles in attenuating psychological distress among married female immigrants [23]. Moreover, unmet healthcare needs is negatively related to their self-rated health [24]. Our study complements previous studies and suggests that one such social determinant of health, divorce, is related to married female immigrants’ access to healthcare. Recognizing the negative association between health and the social determinants of health, and formulating a solution to this problem is a central tenet of public health initiatives.

The association between divorce and access to healthcare services differed according to sex. Married female immigrants exhibited an association between divorce and access to healthcare services, but not married male immigrants. Studies have also reported that access to healthcare services is influenced by socioeconomic status [25]. Finance is a factor that varies greatly depending on the sex of married immigrants. For example, the employment rate of married male immigrants can be significantly higher than that of married female immigrants [10]. Our results also suggest that financial capability affects access to healthcare services. Notably, Korea is currently conducting several projects to increase the employment rate of married female immigrants [26]. By enhancing their financial capability, increased employment rates may be associated with increased access to healthcare services in married female immigrants. Future studies can analyze the current status of the married immigrant policies in South Korea and examine whether the employment rate of married immigrants indeed affects their access to healthcare services.

In preparation for the expansion of a multicultural society, an increasing number of welfare policies (e.g., multicultural family support projects) for married immigrants residing in South Korea are being implemented [27]. Consequently, the experience of social discrimination and depression among married immigrants has declined. However, the use of healthcare services has remained constant or even decreased [9]. This finding suggests an insufficiency of public health policies which are specifically for married immigrants. The role of public health is to prevent diseases and promote health [20]. For social integration, public health should consider the background and environment of the individuals [28]. Divorce status has been a well-studied factor which influences an individual [29]. However, research on the effects of divorce on healthcare utilization is insufficient. To improve the quality of public health, in-depth research on the association between the social determinants of health and healthcare utilization is needed.

A decrease in access to healthcare services can lead to a decrease in health literacy. Research highlights financial capability and language barriers as reasons for the low use of healthcare services by married immigrants [30]. Immigrants’ health literacy is significantly lower than that of ordinary Koreans, with studies showing that lower health literacy is associated with higher dissatisfaction with life [31]. In addition, although married male immigrants have lower health literacy skills than married female immigrants [3], few studies have examined the former’s access to healthcare services. South Korea discloses information about the use of healthcare services and the necessary vocabulary for married immigrants; however, we cannot confirm whether they actually use the disclosed information [32]. In addition, married immigrants still have a high demand for healthcare service education, suggesting the need for direct healthcare education services [33]. Therefore, we recommend including healthcare service education in the social integration programs currently being implemented in South Korea. This can help improve the health literacy of married immigrants and their access to healthcare services.

In this study, selection bias, which is a typical limitation of observational research, was addressed by using propensity score matching. Homogeneity verification indirectly confirmed that the statistically analyzed sample was representative of the population. The heterogeneity of variance should be examined for differences in demographics and confounders across the comparison groups [34]. If the homogeneity of each group is not verified, robust results cannot be obtained when determining the effects of the independent variables on the dependent variable. In the case of experimental studies, the effectiveness of the interventions applied by researchers can be questioned [35]. Here, by using propensity score matching methods, we analyzed the association between divorce and access to healthcare services while securing the homogeneity of all demographic characteristics and confounders across comparison groups. We found that access to healthcare services was affected by several factors (e.g., financial support and economic problems). In our analysis, we controlled for variables related to access to healthcare services. The results were robust and divorce was found to be associated with access to healthcare services even after controlling for other variables.

## Limitations

This study has some limitations. First, while various statistical models were utilized to control for selection bias, accurate causal inferences could not be made in this cross-sectional study. In other words, although divorce was associated with the use of healthcare services among married immigrants, it is unclear whether divorce directly restricts access to healthcare. Future studies should examine the causal relationships between divorce and access to healthcare services. Second, the study database did not contain additional factors that may be associated with the use of healthcare services by married immigrants (e.g., occupation or chronic condition). Future studies should verify the study findings by including various health behaviors. Third, among the immigrant types, we focused on healthcare access of married immigrants. Clear participant selection helps in understanding their characteristics; however, as immigrant types vary, we cannot account for the overall characteristics of immigrants and refugees. Therefore, future research should examine access to healthcare according to the type of immigrants and refugees.

## Conclusion

This study investigated the association between divorce and use of healthcare services among married immigrants. Divorced immigrants had lower access to healthcare services than non-divorced immigrants. Many welfare policies and adaptation programs are in place for married immigrants, but health programs are still in high demand. Furthermore, there is a lack of adaptation programs for vulnerable subpopulations, such as divorced immigrants. This study suggests the need to establish a healthcare or social program to improve health literacy and access to healthcare services for divorced immigrants.

## Data Availability

The datasets analyzed during the current study are available in the Microdata Integrated Service repository https://mdis.kostat.go.kr/index.do.

## Abbreviations

CI: Confidence interval
OR: Odds ratio
IPTW: Inverse probability of treatment weighting
ATE: Average treatment effect
LPS: Logit propensity score
AOR: Adjusted odds ratio
SD: Standard deviation
